# Circulating Sex Hormone Levels and Survival in Men With Esophageal Squamous Cell Carcinoma

**DOI:** 10.1002/cam4.72177

**Published:** 2026-08-17

**Authors:** Zhiqiang Liu, Zhifeng Lin, Jesper Lagergren, Hejie Wang, Xiaodan Mao, Mei Ma, Zhijian Hu, Shao‐Hua Xie

**Affiliations:** ^1^ School of Public Health Fujian Medical University Fuzhou China; ^2^ Section of Esophageal and Mediastinal Oncology, Department of Thoracic Surgery National Cancer Center/National Clinical Research Center for Cancer/Cancer Hospital, Chinese Academy of Medical Sciences and Peking Union Medical College Beijing China; ^3^ Upper Gastrointestinal Surgery, Department of Molecular Medicine and Surgery Karolinska Institutet, Karolinska University Hospital Stockholm Sweden; ^4^ School of Cancer and Pharmaceutical Sciences King's College London London UK; ^5^ Laboratory of Gynecologic Oncology, Fujian Maternity and Child Health Hospital, College of Clinical Medicine for Obstetrics & Gynecology and Pediatrics Fujian Medical University Fuzhou China; ^6^ Department of Laboratory Medicine, Fujian Maternity and Child Health Hospital, College of Clinical Medicine for Obstetrics & Gynecology and Pediatrics Fujian Medical University Fuzhou China; ^7^ Institute of Population Medicine Fujian Medical University Fuzhou China; ^8^ Key Laboratory of Ministry of Education for Gastrointestinal Cancer Fujian Medical University Fuzhou China; ^9^ Fujian Provincial Key Laboratory of Environment Factors and Cancer Fujian Medical University Fuzhou China

**Keywords:** curative surgery, esophageal squamous cell carcinoma, prognosis, sex hormones, survival

## Abstract

**Background:**

Men with esophageal squamous cell carcinoma (ESCC) have worse survival than women, a disparity in which sex hormones may play a role. Yet, whether circulating sex hormone levels are associated with survival in ESCC remains largely unexplored.

**Methods:**

This prospective cohort study included 402 men with ESCC who underwent curative surgery in 2013–2020, with follow‐up until May 1, 2023. Associations between preoperative levels of 11 sex hormone measures and all‐cause mortality were assessed using multivariable Cox regression, reporting hazard ratios (HRs) and 95% confidence intervals (CIs), adjusted for age, calendar year of surgery, education, smoking, alcohol consumption, pathologic tumor stage, postoperative complications, and chemo(radio)therapy. Sex hormone measures were analyzed both as quartiles and continuously. Continuous analyses were conducted using z‐scores of ln‐transformed hormone levels, with HRs interpreted per one standard deviation (SD) increase.

**Results:**

In the overall cohort, no clear associations were observed after adjustment. In tumor stage‐stratified analyses, higher free testosterone index was associated with lower mortality in stage 0–II disease (HR per 1‐SD increase, 0.78; 95% CI, 0.62–0.97), whereas higher progesterone levels were associated with higher mortality in stage III–IV disease (HR per 1‐SD increase, 1.21; 95% CI, 1.01–1.44), with evidence of effect modification by tumor stage for progesterone (*p* for interaction = 0.011).

**Conclusions:**

Preoperative sex hormone measures showed possible stage‐dependent associations with survival in men with ESCC, with higher free testosterone index associated with better survival in stage 0–II disease and higher progesterone levels associated with worse survival in stage III–IV disease.

## 1 Introduction

Esophageal cancer is the 11th most common cancer and the 7th leading cause of cancer deaths worldwide [1]. Esophageal squamous cell carcinoma (ESCC) and esophageal adenocarcinoma are the two main histological subtypes, among which ESCC accounts for approximately 90% of all cases globally and is particularly dominant in Asian countries [2]. Despite advances in diagnosis and treatment, ESCC continues to carry a poor prognosis [3, 4, 5], and tumor stage at diagnosis remains a major determinant of survival [3, 4]. For patients with resectable localized or locally advanced ESCC, esophagectomy, either alone or as part of multimodal treatment, remains a cornerstone of curative‐intent therapy [3].

ESCC is more common among men than women, with the male‐to‐female incidence ratio of 3‐to‐1 globally and up to 8‐to‐1 in Eastern Europe [6]. A protective role of estrogenic and antiandrogenic hormones in the etiology of ESCC has been indicated by a lower risk in users of menopausal hormone therapy and 5α‐reductase inhibitors, respectively [7, 8]. Survival in ESCC is better in women than men, particularly in young patients [9, 10, 11]. This disparity is not explained by differences in known prognostic factors. Differences in circulating sex hormone levels may partly explain the survival advantage observed in women. Although circulating sex hormone levels have been investigated in relation to survival in esophageal adenocarcinoma [12], evidence on their association with survival in ESCC remains limited.

This study aimed to investigate the associations between preoperative circulating sex hormone measures and all‐cause mortality in men undergoing curative surgery for ESCC.

## 2 Methods

### 2.1 Study Design and Participants

This prospective cohort study included 402 newly diagnosed male patients with histologically confirmed primary ESCC who had undergone surgery with curative intent. We did not include female patients due to the limited number. These participants were recruited from the First Affiliated Hospital and Zhangzhou Affiliated Hospital of Fujian Medical University and Fujian Provincial Cancer Hospital during the 7‐year period from November 1, 2013 to November 30, 2020. All participants were followed up via telephone calls by research staff every 3 months during the first year after surgery and every 6 months thereafter. The follow‐up ended at death or the end of the study period (May 1, 2023), whichever occurred first. Local cause‐of‐death registries were queried to check the vital status of patients who were lost to telephone follow‐up. Patients were censored on the date of last contact if no death record was identified in the death registry. The study was approved by the Ethics Committee of Fujian Medical University (reference no. 201495), and all participants signed informed consent forms.

### 2.2 Sex Hormone Exposures

Upon admission to the hospital, each patient eligible for inclusion was invited to donate 5 mL of peripheral blood for this study. For all participants, plasma samples were obtained after centrifugation (3,000 rpm for 15 min at room temperature) and stored at −80°C until analysis. The plasma levels of the following 8 sex hormone measures were analyzed: Sex hormone‐binding globulin (SHBG), testosterone, estradiol, progesterone, follicle‐stimulating hormone, luteinizing hormone, prolactin, and dehydroepiandrosterone sulfate (DHEAS). We also calculated the free testosterone index (testosterone × 10/SHBG), the free estradiol index (estradiol × 10/SHBG), and the testosterone:estradiol ratio. These 11 hormonal measures in total cover key points in the biosynthesis of sex hormones [13, 14, 15]. Levels of SHBG, testosterone, estradiol, progesterone, follicle‐stimulating hormone, luteinizing hormone, prolactin, and DHEAS were measured using chemiluminescent microparticle immunoassay (CMIA) on the ARCHITECT i2000SR immunoassay analyzer (Abbott, Illinois, the United States), following standard laboratory protocols. Each batch included blinded quality‐control replicates. Within‐ and between‐batch coefficients of variation were < 5%, and the intraclass correlation coefficient was > 0.86 for all hormone measures. We also assessed changes in sex hormone levels before and after surgery in 53 patients from whom paired blood samples were collected. The postoperative sample was scheduled to be collected on postoperative day 7 (±2 days).

### 2.3 Mortality Outcome

The outcome was all‐cause mortality, defined as death due to any cause occurring after ESCC surgery.

### 2.4 Covariates

The covariates were the following 8 known or potential prognostic factors: Age at surgery (continuous); calendar year of surgery (continuous); educational level (categorized as primary school or below, secondary school, or college or above); tobacco smoking (yes or no, defined as daily smoking for at least 6 months or cumulative smoking of at least 150 cigarettes before diagnosis); alcohol consumption (yes or no, defined as at least weekly for 6 months before diagnosis); pathologic tumor stage (pTNM 0–II or III–IV according to the 8th edition of Union for International Cancer Control/American Joint Committee on Cancer staging of esophageal cancer); in‐hospital postoperative complications (yes or no); and neoadjuvant or adjuvant chemo(radio)therapy (yes or no). Information on sociodemographic characteristics and lifestyle factors was obtained from structured interviews conducted before surgery, and clinical information was retrieved by reviewing medical records.

### 2.5 Statistical Analysis

Overall survival probabilities at 1, 3, and 5 years were estimated using the Kaplan–Meier method. Pairwise Spearman correlation analysis was used to assess correlations among the 11 studied sex hormone measures. Associations between preoperative sex hormone measures and all‐cause mortality were assessed using Cox proportional hazards regression, reporting hazard ratios (HRs) and 95% confidence intervals (CIs). Sex hormone measures were analyzed both as quartiles and on a continuous scale. Quartiles were defined using the original (untransformed) measurements; HRs compared quartiles 2–4 with quartile 1. *p* for trend was derived by modeling quartile rank (1–4) as a continuous variable in the corresponding Cox model. Continuous analyses were performed using ln‐transformed hormone levels, which were then standardized to z‐scores [(ln(value) − mean)/SD]; HRs were interpreted per one standard deviation (SD) increase. For ratios and indices, the ratio/index was calculated first and then ln‐transformed and z‐standardized. Three models were fitted: An unadjusted model; a basic model adjusted for age and calendar year of surgery; and a fully adjusted model additionally adjusted for educational level, tobacco smoking, alcohol consumption, pathologic tumor stage, in‐hospital postoperative complications, and chemo(radio)therapy. Stage‐stratified analyses (0–II vs. III–IV) were performed, and multiplicative interactions were evaluated based on the statistical significance of the cross‐product term between tumor stage and the continuous (ln‐transformed, z‐standardized) hormone variable. To assess the potential influence of patients lost to follow‐up, two sensitivity analyses were conducted assuming that these patients (i) died on the date of last contact or (ii) were alive by the end of the study period; these sensitivity analyses were applied to both quartile‐based and continuous‐scale models. In the 53 patients with paired preoperative and postoperative blood samples, paired *t*‐tests were performed on ln‐transformed values to compare preoperative and postoperative hormone levels. All statistical analyses were performed using R software (version 4.5.1; R Foundation for Statistical Computing, Vienna, Austria). All tests were two‐sided, and statistical significance was defined as *p* < 0.05.

## 3 Results

### 3.1 Participants

Baseline characteristics of the 402 participants are presented in Table 1. The average age at surgery was 59.7 (±SD 8.2) years. Of these patients, 213 (53.0%) were diagnosed at pathologic stages III–IV, 177 (44.0%) experienced in‐hospital postoperative complications, and 158 (39.3%) received neoadjuvant or adjuvant chemo(radio)therapy. During a median follow‐up of 63 months, 195 (48.5%) patients died. Fifty‐four (13.4%) patients were lost to follow‐up, and these were more likely to have tumors at advanced stages and in‐hospital postoperative complications compared with the other patients in the cohort (Table 1). In the overall cohort, the 1‐, 3‐, and 5‐year survival rates were 84.2%, 54.8%, and 43.7%, respectively.

**TABLE 1:** Baseline characteristics of 402 men with esophageal squamous cell carcinoma (ESCC) undergoing curative surgery, by follow‐up status.

Characteristics	Total, Number (%)	Not lost to follow‐up, Number (%)	Lost to follow‐up, Number (%)
Number of participants	402 (100.0)	348 (86.6)	54 (13.4)
Age at surgery, years			
< 60	185 (46.0)	156 (44.8)	29 (53.7)
60 to 69	175 (43.5)	156 (44.8)	19 (35.2)
≥ 70	42 (10.4)	36 (10.3)	6 (11.1)
Mean ± SD	59.7 ± 8.2	59.8 ± 8.3	58.9 ± 7.6
Year of surgery			
2013 to 2016	169 (42.0)	134 (38.5)	35 (64.8)
2017 to 2020	233 (58.0)	214 (61.5)	19 (35.2)
Educational level			
Primary school or below	235 (58.5)	202 (58.0)	33 (61.1)
Secondary school	155 (38.6)	136 (39.1)	19 (35.2)
College or above	12 (3.0)	10 (2.9)	2 (3.7)
Tobacco smoking			
No	47 (11.7)	40 (11.5)	7 (13.0)
Yes	355 (88.3)	308 (88.5)	47 (87.0)
Alcohol consumption			
No	140 (34.8)	117 (33.6)	23 (42.6)
Yes	262 (65.2)	231 (66.4)	31 (57.4)
Pathologic tumor stage			
0–II	189 (47.0)	168 (48.3)	21 (38.9)
III–IV	213 (53.0)	180 (51.7)	33 (61.1)
In‐hospital postoperative complications			
No	225 (56.0)	204 (58.6)	21 (38.9)
Yes	177 (44.0)	144 (41.4)	33 (61.1)
Chemo(radio)therapy			
No	244 (60.7)	212 (60.9)	32 (59.3)
Yes	158 (39.3)	136 (39.1)	22 (40.7)

*Note:* Values are *n* (%) unless otherwise indicated. Age is presented as mean ± standard deviation (SD).

### 3.2 Sex Hormone Measures

The distribution of circulating levels of each sex hormone measure before surgery is shown in Table S1. The strongest correlations were found for the pairings of testosterone and the testosterone:estradiol ratio (0.85), SHBG and the free estradiol index (−0.77), testosterone and the free testosterone index (0.69), follicle‐stimulating hormone and luteinizing hormone (0.66), free estradiol index and the testosterone:estradiol ratio (−0.64), SHBG and testosterone (0.61), and free testosterone index and testosterone:estradiol ratio (0.60), all with *p* < 0.001 (Figure S1).

Among the 53 patients with paired blood samples, the postoperative sample was collected at a median of 7 days after surgery (interquartile range 5–8 days). In paired *t*‐tests on ln‐transformed concentrations, postoperative levels were significantly lower for SHBG, testosterone, free testosterone index, and the testosterone:estradiol ratio (all *p* < 0.001), and significantly higher for estradiol (*p* < 0.001), free estradiol index (*p* < 0.001) and prolactin (*p* = 0.017); no statistically significant differences were observed for DHEAS, follicle‐stimulating hormone, luteinizing hormone, or progesterone (Table S2).

### 3.3 Risk of All‐Cause Mortality

Higher free testosterone index was associated with lower mortality in the crude model, but the association was attenuated after full adjustment (HR 0.74, 95% CI 0.47–1.15, 4th vs. 1st quartile). In the continuous analysis, the fully adjusted HR per 1‐SD increase in ln‐transformed free testosterone index was 0.88 (95% CI 0.76–1.02). Higher levels of prolactin were associated with a possibly higher risk of mortality in quartile analyses (fully adjusted HR 1.50, 95% CI 0.99–2.28, 4th vs. 1st quartile) and in the continuous analysis (per 1‐SD increase: HR 1.14, 95% CI 0.99–1.31). No clear associations were found for any of the other studied sex hormone measures, i.e., SHBG, testosterone, DHEAS, estradiol, free estradiol index, testosterone:estradiol ratio, follicle‐stimulating hormone, luteinizing hormone, and progesterone (Table 2).

**TABLE 2:** Associations between preoperative circulating levels of sex hormone measures and all‐cause mortality in men with esophageal squamous cell carcinoma.

Sex hormone	Number of participants	Unadjusted model HR (95% CI)	Basic model HR (95% CI)^a^	Full model HR (95% CI)^b^
Sex hormone‐binding globulin, nmol/L				
< 35.30	94	1.00 (reference)	1.00 (reference)	1.00 (reference)
35.30 to < 45.70	93	1.45 (0.95, 2.20)	1.40 (0.92, 2.14)	1.47 (0.96, 2.25)
45.70 to < 58.45	96	1.35 (0.89, 2.04)	1.32 (0.87, 2.01)	1.40 (0.91, 2.14)
≥ 58.45	94	1.33 (0.87, 2.04)	1.23 (0.80, 1.89)	1.29 (0.83, 2.00)
*p* for trend		0.255	0.457	0.339
Continuous, per 1‐SD increase^c^	377	1.16 (1.00, 1.35)	1.12 (0.97, 1.30)	1.14 (0.98, 1.32)
Testosterone, nmol/L				
< 11.35	99	1.00 (reference)	1.00 (reference)	1.00 (reference)
11.35 to < 18.68	100	0.85 (0.57, 1.27)	0.87 (0.58, 1.30)	0.96 (0.64, 1.45)
18.68 to < 24.72	100	0.74 (0.50, 1.10)	0.75 (0.50, 1.13)	0.83 (0.55, 1.26)
≥ 24.72	100	0.83 (0.56, 1.24)	0.84 (0.56, 1.25)	1.02 (0.67, 1.53)
*p* for trend		0.288	0.305	0.873
Continuous, per 1‐SD increase^c^	399	0.96 (0.83, 1.10)	0.96 (0.83, 1.11)	1.01 (0.87, 1.17)
Free testosterone index				
< 2.68	93	1.00 (reference)	1.00 (reference)	1.00 (reference)
2.68 to < 3.89	94	0.92 (0.62, 1.36)	0.85 (0.57, 1.27)	0.92 (0.61, 1.39)
3.89 to < 4.85	94	0.70 (0.47, 1.05)	0.65 (0.43, 0.98)	0.76 (0.50, 1.15)
≥ 4.85	93	0.65 (0.43, 0.98)	0.66 (0.43, 1.02)	0.74 (0.47, 1.15)
*p* for trend		0.017	0.025	0.116
Continuous, per 1‐SD increase^c^	374	0.84 (0.74, 0.96)	0.84 (0.73, 0.97)	0.88 (0.76, 1.02)
Dehydroepiandrosterone sulfate, μg/dL				
< 96.30	99	1.00 (reference)	1.00 (reference)	1.00 (reference)
96.30 to < 131.75	100	0.95 (0.64, 1.40)	0.96 (0.65, 1.43)	0.98 (0.66, 1.46)
131.75 to < 188.80	100	1.06 (0.72, 1.55)	1.10 (0.75, 1.62)	1.29 (0.87, 1.91)
≥ 188.80	99	0.67 (0.44, 1.02)	0.74 (0.48, 1.14)	0.82 (0.53, 1.26)
*p* for trend		0.112	0.311	0.702
Continuous, per 1‐SD increase^c^	398	0.88 (0.76, 1.01)	0.91 (0.79, 1.05)	0.94 (0.81, 1.09)
Estradiol, nmol/L				
< 0.106	78	1.00 (reference)	1.00 (reference)	1.00 (reference)
0.106 to < 0.132	95	0.80 (0.52, 1.23)	0.76 (0.49, 1.17)	0.82 (0.53, 1.26)
0.132 to < 0.154	88	0.77 (0.49, 1.20)	0.73 (0.47, 1.15)	0.81 (0.51, 1.28)
≥ 0.154	91	0.89 (0.57, 1.37)	0.83 (0.53, 1.29)	0.82 (0.52, 1.28)
*p* for trend		0.637	0.473	0.434
Continuous, per 1‐SD increase^c^	352	0.97 (0.82, 1.14)	0.93 (0.79, 1.10)	0.95 (0.81, 1.12)
Free estradiol index				
< 0.021	83	1.00 (reference)	1.00 (reference)	1.00 (reference)
0.021 to < 0.028	83	1.00 (0.65, 1.54)	1.03 (0.67, 1.59)	1.02 (0.66, 1.58)
0.028 to < 0.038	81	0.98 (0.63, 1.51)	0.97 (0.63, 1.50)	1.05 (0.68, 1.65)
≥ 0.038	83	0.73 (0.46, 1.16)	0.78 (0.49, 1.24)	0.74 (0.46, 1.20)
*p* for trend		0.197	0.288	0.274
Continuous, per 1‐SD increase^c^	330	0.88 (0.75, 1.03)	0.89 (0.76, 1.05)	0.89 (0.75, 1.05)
Testosterone:estradiol ratio				
< 86.04	87	1.00 (reference)	1.00 (reference)	1.00 (reference)
86.04 to < 145.28	87	0.89 (0.58, 1.36)	0.93 (0.61, 1.43)	0.98 (0.64, 1.52)
145.28 to < 185.76	88	0.77 (0.50, 1.18)	0.79 (0.51, 1.22)	0.76 (0.49, 1.18)
≥ 185.76	87	0.69 (0.45, 1.08)	0.73 (0.46, 1.15)	0.85 (0.53, 1.35)
*p* for trend		0.077	0.119	0.284
Continuous, per 1‐SD increase^c^	349	0.96 (0.82, 1.11)	0.98 (0.84, 1.14)	1.01 (0.86, 1.19)
Prolactin, ng/mL				
< 10.55	95	1.00 (reference)	1.00 (reference)	1.00 (reference)
10.55 to < 13.74	96	1.25 (0.82, 1.90)	1.22 (0.80, 1.87)	1.22 (0.80, 1.87)
13.74 to < 19.56	96	1.09 (0.71, 1.69)	1.08 (0.70, 1.68)	1.00 (0.65, 1.56)
≥ 19.56	96	1.59 (1.05, 2.39)	1.57 (1.04, 2.37)	1.50 (0.99, 2.28)
*p* for trend		0.055	0.058	0.122
Continuous, per 1‐SD increase^c^	383	1.17 (1.02, 1.34)	1.16 (1.01, 1.33)	1.14 (0.99, 1.31)
Follicle‐stimulating hormone, mIU/mL				
< 5.77	98	1.00 (reference)	1.00 (reference)	1.00 (reference)
5.77 to < 8.28	99	1.16 (0.77, 1.75)	1.07 (0.71, 1.62)	1.10 (0.73, 1.67)
8.28 to < 12.23	99	1.25 (0.84, 1.87)	1.15 (0.77, 1.73)	1.13 (0.75, 1.69)
≥ 12.23	98	1.09 (0.72, 1.65)	0.88 (0.57, 1.37)	0.83 (0.53, 1.28)
*p* for trend		0.620	0.673	0.441
Continuous, per 1‐SD increase^c^	394	1.06 (0.92, 1.22)	0.99 (0.85, 1.15)	0.96 (0.82, 1.11)
Luteinizing hormone, mIU/mL				
< 3.55	98	1.00 (reference)	1.00 (reference)	1.00 (reference)
3.55 to < 5.24	97	0.74 (0.49, 1.11)	0.71 (0.47, 1.07)	0.82 (0.54, 1.25)
5.24 to < 7.26	100	0.96 (0.65, 1.41)	0.91 (0.61, 1.35)	0.98 (0.66, 1.46)
≥ 7.26	98	0.94 (0.63, 1.40)	0.79 (0.53, 1.20)	0.82 (0.54, 1.24)
*p* for trend		0.918	0.510	0.534
Continuous, per 1‐SD increase^c^	393	0.95 (0.82, 1.10)	0.89 (0.77, 1.03)	0.90 (0.78, 1.04)
Progesterone, ng/mL				
< 0.11	104	1.00 (reference)	1.00 (reference)	1.00 (reference)
0.11 to < 0.16	88	0.96 (0.63, 1.44)	0.92 (0.61, 1.39)	0.93 (0.61, 1.41)
0.16 to < 0.22	97	1.13 (0.76, 1.68)	1.20 (0.80, 1.78)	1.49 (0.99, 2.25)
≥ 0.22	88	0.96 (0.63, 1.45)	0.98 (0.64, 1.48)	1.12 (0.73, 1.71)
*p* for trend		0.945	0.766	0.244
Continuous, per 1‐SD increase^c^	377	1.04 (0.88, 1.23)	1.06 (0.90, 1.25)	1.09 (0.93, 1.28)

Abbreviations: CI, Confidence interval; HR, Hazard ratio.

^a^ Adjusted for age and calendar year of surgery.

^b^ Adjusted for age and calendar year of surgery, educational level, tobacco smoking, alcohol consumption, pathologic tumor stage, in‐hospital postoperative complications, and chemo(radio)therapy.

^c^ Hazard ratio per one standard deviation (SD) increase in ln‐transformed hormone levels. *p* for trend was derived by modeling quartile rank (1–4) as a continuous variable.

When stratified by tumor stage (Table 3), higher free testosterone index showed an inverse association with mortality among men with stage 0–II tumors (per 1‐SD increase: HR 0.78, 95% CI 0.62–0.97; *p* for trend = 0.054), whereas no clear association was observed in stage III–IV tumors (*p* for interaction = 0.105). In contrast, among men with stage III–IV tumors, higher progesterone levels were associated with higher mortality (per 1‐SD increase: HR 1.21, 95% CI 1.01–1.44; *p* for trend = 0.016), with evidence of effect modification by tumor stage (*p* for interaction = 0.011).

**TABLE 3:** Associations between preoperative circulating levels of sex hormone measures and all‐cause mortality in men with esophageal squamous cell carcinoma, stratified by pathologic tumor stage.

Sex hormone	Stages 0–II		Stages III–IV	
	Number of participants	HR (95% CI)^a^	Number of participants	HR (95% CI)^a^
Sex hormone‐binding globulin, nmol/L				
< 35.30	40	1.00 (reference)	54	1.00 (reference)
35.30 to < 45.70	48	2.10 (0.98, 4.49)	45	1.28 (0.76, 2.17)
45.70 to < 58.45	48	1.68 (0.77, 3.66)	48	1.25 (0.74, 2.09)
≥ 58.45	44	1.46 (0.65, 3.27)	50	1.29 (0.76, 2.21)
*p* for trend		0.613		0.383
Continuous, per 1‐SD increase^b^	180	1.12 (0.87, 1.44)	197	1.16 (0.96, 1.40)
*p* for interaction = 0.911				
Testosterone, nmol/L				
< 11.35	43	1.00 (reference)	56	1.00 (reference)
11.35 to < 18.68	42	0.48 (0.23, 1.04)	58	1.15 (0.70, 1.88)
18.68 to < 24.72	50	0.68 (0.35, 1.32)	50	0.85 (0.49, 1.45)
≥ 24.72	52	0.55 (0.27, 1.12)	48	1.34 (0.80, 2.22)
*p* for trend		0.244		0.519
Continuous, per 1‐SD increase^b^	187	0.88 (0.70, 1.11)	212	1.10 (0.91, 1.35)
*p* for interaction = 0.111				
Free testosterone index				
< 2.68	38	1.00 (reference)	55	1.00 (reference)
2.68 to < 3.89	44	0.88 (0.44, 1.76)	50	0.87 (0.51, 1.47)
3.89 to < 4.85	50	0.49 (0.24, 0.99)	44	0.92 (0.55, 1.56)
≥ 4.85	47	0.59 (0.28, 1.26)	46	0.77 (0.44, 1.36)
*p* for trend		0.054		0.444
Continuous, per 1‐SD increase^b^	179	0.78 (0.62, 0.97)	195	0.93 (0.76, 1.13)
*p* for interaction = 0.105				
Dehydroepiandrosterone sulfate, μg/dL				
< 96.30	42	1.00 (reference)	57	1.00 (reference)
96.30 to < 131.75	47	0.79 (0.39, 1.60)	53	1.03 (0.64, 1.67)
131.75 to < 188.80	47	1.24 (0.63, 2.43)	53	1.27 (0.77, 2.10)
≥ 188.80	50	0.69 (0.33, 1.45)	49	0.83 (0.48, 1.43)
*p* for trend		0.605		0.741
Continuous, per 1‐SD increase^b^	186	0.93 (0.72, 1.20)	212	0.93 (0.78, 1.12)
*p* for interaction = 0.720				
Estradiol, nmol/L				
< 0.106	32	1.00 (reference)	46	1.00 (reference)
0.106 to < 0.132	51	1.52 (0.69, 3.34)	44	0.58 (0.32, 1.05)
0.132 to < 0.154	44	0.91 (0.37, 2.22)	44	0.83 (0.47, 1.45)
≥ 0.154	39	1.44 (0.62, 3.32)	52	0.66 (0.38, 1.14)
*p* for trend		0.804		0.324
Continuous, per 1‐SD increase^b^	166	1.00 (0.77, 1.30)	186	0.93 (0.76, 1.14)
*p* for interaction = 0.503				
Free estradiol index				
< 0.021	36	1.00 (reference)	47	1.00 (reference)
0.021 to < 0.028	41	1.04 (0.50, 2.20)	42	1.00 (0.58, 1.73)
0.028 to < 0.038	48	1.41 (0.70, 2.83)	33	0.78 (0.43, 1.41)
≥ 0.038	34	0.64 (0.25, 1.59)	49	0.82 (0.46, 1.45)
*p* for trend		0.668		0.372
Continuous, per 1‐SD increase^b^	159	0.94 (0.73, 1.22)	171	0.88 (0.71, 1.09)
*p* for interaction = 0.724				
Testosterone:estradiol ratio				
< 86.04	41	1.00 (reference)	46	1.00 (reference)
86.04 to < 145.28	37	0.77 (0.37, 1.60)	50	1.08 (0.62, 1.87)
145.28 to < 185.76	36	0.62 (0.30, 1.31)	52	0.84 (0.48, 1.45)
≥ 185.76	51	0.50 (0.25, 1.04)	36	1.23 (0.67, 2.24)
*p* for trend		0.051		0.850
Continuous, per 1‐SD increase^b^	165	0.89 (0.71, 1.11)	184	1.13 (0.91, 1.41)
*p* for interaction = 0.103				
Prolactin, ng/mL				
< 10.55	49	1.00 (reference)	46	1.00 (reference)
10.55 to < 13.74	44	1.04 (0.52, 2.11)	52	1.30 (0.75, 2.27)
13.74 to < 19.56	44	1.00 (0.48, 2.07)	52	1.10 (0.62, 1.94)
≥ 19.56	44	1.56 (0.82, 2.98)	52	1.46 (0.83, 2.56)
*p* for trend		0.203		0.304
Continuous, per 1‐SD increase^b^	181	1.24 (0.99, 1.55)	202	1.08 (0.90, 1.30)
*p* for interaction = 0.427				
Follicle‐stimulating hormone, mIU/mL				
< 5.77	49	1.00 (reference)	49	1.00 (reference)
5.77 to < 8.28	48	0.95 (0.47, 1.92)	51	1.24 (0.73, 2.10)
8.28 to < 12.23	45	1.24 (0.64, 2.41)	54	1.12 (0.67, 1.88)
≥ 12.23	44	0.82 (0.39, 1.70)	54	0.83 (0.48, 1.45)
*p* for trend		0.807		0.464
Continuous, per 1‐SD increase^b^	186	0.97 (0.77, 1.24)	208	0.96 (0.79, 1.16)
*p* for interaction = 0.820				
Luteinizing hormone, mIU/mL				
< 3.55	43	1.00 (reference)	55	1.00 (reference)
3.55 to < 5.24	52	0.75 (0.38, 1.49)	45	0.80 (0.46, 1.37)
5.24 to < 7.26	50	0.98 (0.50, 1.91)	50	0.95 (0.58, 1.56)
≥ 7.26	41	0.83 (0.40, 1.72)	57	0.82 (0.49, 1.36)
*p* for trend		0.866		0.579
Continuous, per 1‐SD increase^b^	186	0.92 (0.72, 1.19)	207	0.90 (0.75, 1.07)
*p* for interaction = 0.753				
Progesterone, ng/mL				
< 0.11	43	1.00 (reference)	61	1.00 (reference)
0.11 to < 0.16	42	1.27 (0.66, 2.46)	46	0.73 (0.42, 1.28)
0.16 to < 0.22	51	0.83 (0.41, 1.67)	46	2.09 (1.25, 3.48)
≥ 0.22	43	0.69 (0.33, 1.47)	45	1.46 (0.86, 2.47)
*p* for trend		0.186		0.016
Continuous, per 1‐SD increase^b^	179	0.78 (0.56, 1.09)	198	1.21 (1.01, 1.44)
*p* for interaction = 0.011				

Abbreviations: CI, Confidence interval; HR, Hazard ratio.

^a^ Adjusted for age and calendar year of surgery, educational level, tobacco smoking, alcohol consumption, in‐hospital postoperative complications, and chemo(radio)therapy.

^b^ Hazard ratio per one standard deviation (SD) increase in ln‐transformed hormone levels. *p* for trend was derived by modeling quartile rank (1–4) as a continuous variable. *p* for interaction was obtained from the cross‐product term between pathologic tumor stage (0–II vs. III–IV) and each continuous (ln‐transformed, z‐standardized) hormone variable in the Cox model.

Sensitivity analyses assuming that all participants lost to follow‐up either died on the date of last contact or were alive by the end of the study period showed similar patterns for both quartile‐based and continuous‐scale analyses (Table S3).

## 4 Discussion

This study did not identify clear overall associations between preoperative circulating levels of 11 sex hormone measures and all‐cause mortality among men undergoing curative surgery for ESCC. However, in stage‐stratified analyses, higher free testosterone index was associated with lower mortality in stage 0–II disease, whereas higher progesterone levels were associated with higher mortality in stage III–IV disease, with evidence of effect modification by tumor stage for progesterone. No clear stage‐specific associations were observed for the remaining sex hormone measures.

Strengths of this study include the prospective design, assessment of a comprehensive panel of sex hormones using up‐to‐date assays, and adjustment for major prognostic factors. Several limitations should be noted. First, sex hormone levels were measured in a single preoperative blood sample and may not reflect long‐term exposure; moreover, the presence of cancer may have influenced circulating hormone levels. Second, this study was restricted to men because few women were available in our cohort and because sex hormone measurements in women are strongly influenced by menstrual cycle variation and reproductive factors. This restriction limits the generalizability of our findings to women and precludes direct evaluation of sex‐specific differences. Accordingly, our findings should be interpreted as applying specifically to men undergoing curative surgery for ESCC. Third, more than 10% of participants were lost to follow‐up, which may introduce selection bias. However, sensitivity analyses using worst‐case and best‐case assumptions regarding their vital status suggested that substantial selection bias is unlikely. Finally, given multiple testing and the limited sample size, some findings may be due to chance.

To the best of our knowledge, this study was the first to prospectively investigate associations between preoperative circulating sex hormone levels and survival after curative surgery for ESCC. The “sex hormone hypothesis” suggests that higher estrogen levels and lower testosterone levels may be associated with a lower risk of ESCC and be associated with better survival. This hypothesis has received some support from experimental evidence showing a growth‐promoting effect of testosterone in ESCC cell lines [16] and from studies implicating androgen receptor signaling in ESCC progression and prognosis [17, 18, 19]. However, a Swedish population‐based cohort study did not find evidence that use of the anti‐androgenic drug 5α‐reductase inhibitors was associated with lower 5‐year all‐cause mortality among patients with ESCC [20]. In contrast to the hypothesis, our stage‐stratified analyses showed that a higher free testosterone index might be associated with lower all‐cause mortality, particularly in patients with stage 0–II disease. Overall, the existing evidence regarding the role of testosterone in ESCC survival remains uncertain, and the underlying biological mechanisms remain largely unclear.

It should be noted that circulating estradiol levels may not fully reflect local concentrations within the tumor microenvironment. In peripheral tissues, aromatase converts testosterone to estradiol, and this local conversion can generate high tissue estradiol concentrations without significantly affecting circulating levels [21]. Clinical studies in breast cancer have shown that tissue estrogen concentrations are several‐fold higher than those in plasma [22]. Immunohistochemical studies have demonstrated that estrogen receptors are expressed in ESCC tissues [23]. Furthermore, in vitro studies have shown that estradiol inhibits ESCC cell proliferation [24], and pharmacological activation of G protein‐coupled estrogen receptor 1 (GPER1) inhibits proliferation and promotes apoptosis in a GPER1‐positive ESCC cell line [25]. In contrast, androgens promote tumor growth through the androgen receptor [17]. It is therefore plausible that men with a higher free testosterone index may have a better prognosis partly because they have more substrate available for local aromatization to estradiol within the tumor microenvironment. This hypothesis warrants further investigation.

This study suggested that higher circulating prolactin levels were possibly associated with higher mortality in men with ESCC. Previous studies have associated hyperprolactinemia with an increased risk of upper gastrointestinal cancer and tumor prolactin expression with poorer survival in ESCC [26, 27]. Higher circulating prolactin levels have been associated with poorer prognosis in breast cancer, while tumor prolactin expression has been associated with poorer outcomes in breast and endometrial cancers [28, 29]. Prolactin is best known for its role in lactation, but it also participates in immune regulation and angiogenesis [30]. Laboratory studies suggest that prolactin may influence cancer initiation and progression through multiple mechanisms [31, 32], for example by promoting the survival and migration of ovarian cancer cells [33] and by protecting breast cancer cells against apoptosis [34]. However, the role of prolactin in ESCC prognosis and the underlying mechanisms remain unclear.

In stage‐stratified analyses, higher progesterone levels were associated with higher all‐cause mortality among men with advanced‐stage ESCC. Progesterone plays roles in male reproductive physiology and steroidogenesis and exerts its effects primarily through the progesterone receptor [35]. However, evidence on progesterone receptor expression in ESCC is limited; an early study including 30 ESCC patients reported no progesterone receptor expression in normal esophageal mucosa or tumor tissues [36]. In women, menopausal hormone therapy has been associated with a lower risk of ESCC, although it remains unclear whether this association is specific to progestogen‐containing regimens [37, 38]. Yet, exposure to exogenous progestogens in menopausal hormone therapy is not directly comparable to endogenous circulating progesterone levels, and these studies addressed ESCC risk in women rather than survival in men. Thus, whether progesterone plays a role in ESCC survival remains unclear.

Despite advances in multimodal treatment for ESCC, prognosis remains poor [3, 4, 5]. Our findings suggest several directions for future research. First, whether preoperative sex hormone levels are associated with prognosis requires validation in independent, large‐scale prospective studies including sufficient numbers of both men and women to allow direct evaluation of sex‐specific differences. Second, if future research confirms the observed stage‐specific associations for free testosterone index and progesterone, these measures could be further explored as prognostic or predictive biomarkers and as indicators of underlying tumor or host biology. Third, studies with repeated hormone measurements from before diagnosis through treatment and follow‐up would help clarify the temporal dynamics of sex hormones in ESCC and assess whether modulation of hormonal pathways might ultimately have therapeutic implications. At present, our findings do not support modifying treatment strategies based on circulating sex hormone levels.

## 5 Conclusion

In summary, this prospective cohort study found no clear overall associations between preoperative circulating sex hormone measures and all‐cause mortality in men with ESCC undergoing curative surgery. However, stage‐specific patterns were observed: Higher free testosterone index was associated with better survival in stage 0–II disease, whereas higher progesterone levels were associated with worse survival in stage III–IV disease, with evidence of effect modification by tumor stage for progesterone.

## Author Contributions

**Zhiqiang Liu:** conceptualization, data curation, formal analysis, funding acquisition, investigation, methodology, project administration, software, visualization, writing – original draft, writing – review and editing. **Zhifeng Lin:** data curation, investigation, validation. **Jesper Lagergren:** supervision, writing – review and editing. **Hejie Wang:** data curation, investigation. **Xiaodan Mao:** investigation, resources. **Mei Ma:** investigation, resources. **Zhijian Hu:** funding acquisition, methodology, project administration, resources, supervision, writing – review and editing. **Shao‐Hua Xie:** conceptualization, funding acquisition, methodology, project administration, resources, supervision, visualization, writing – review and editing.

## Funding

This study was supported by the Swedish Cancer Society (222038 and 254478), the Scientific Foundation of Fuzhou City (2020‐WS‐57), the Startup Fund for Scientific Research, Fujian Medical University (2021QH1108), and the Natural Science Foundation of Fujian Province (2021J01726 and 2021J01733). The funders had no role in the study design, the collection, analysis, and interpretation of data, or the writing of the article and the decision to submit it for publication.

## Ethics Statement

This study was approved by the Ethics Committee of Fujian Medical University (reference no. 201495). Written informed consent was obtained from all participants before enrollment. The study was conducted in accordance with the Declaration of Helsinki.

## Conflicts of Interest

The authors declare no conflicts of interest.

## Supporting information


**Table S1:** Distributions of preoperative circulating levels of sex hormone measures in men with esophageal squamous cell carcinoma.
**Table S2:** Paired *t*‐tests comparing ln‐transformed preoperative and postoperative circulating levels of sex hormone measures in 53 men with esophageal squamous cell carcinoma.
**Table S3:** Sensitivity analyses of associations between preoperative circulating levels of sex hormone measures and all‐cause mortality in men with esophageal squamous cell carcinoma.
**Figure S1:** Heatmap of pairwise Spearman correlation coefficients among preoperative circulating sex hormone measures in men with esophageal squamous cell carcinoma undergoing curative surgery. SHBG, sex hormone‐binding globulin; T, testosterone; FTI, free testosterone index; DHEAS, dehydroepiandrosterone sulfate; E2, estradiol; FEI, free estradiol index; T/E2, testosterone:estradiol ratio; FSH, follicle‐stimulating hormone; LH, luteinizing hormone; P4, progesterone. Cells display Spearman's ρ values; asterisks indicate statistical significance (*p < 0.05, **p < 0.01, ***p < 0.001).

## Data Availability

The data that support the findings of this study are available from the corresponding author upon reasonable request.
