# Supplementary material for: Circulating Sex Hormone Levels and Survival in Men With Esophageal Squamous Cell Carcinoma
Source: Cancer Med. 2026 Aug 17;15(8):e72177. doi: 10.1002/cam4.72177 (PMC13482017; doi:10.1002/cam4.72177)
Supplement: Supplementary file 1 [file CAM4-15-e72177-s001.pdf]

Circulating Sex Hormone Levels and Survival in Men with Esophageal Squamous Cell Carcinoma

Zhiqiang Liu, Zhifeng Lin, Jesper Lagergren, Hejie Wang, Xiaodan Mao, Mei Ma, Zhijian Hu, Shao-Hua Xie

Supplementary Material

Supplementary Table 1. Distributions of preoperative circulating levels of sex hormone measures in men with esophageal squamous cell carcinoma

Supplementary Table 2. Paired *t*-tests comparing ln-transformed preoperative and postoperative circulating levels of sex hormone measures in 53 men with esophageal squamous cell carcinoma

Supplementary Table 3. Sensitivity analyses of associations between preoperative circulating levels of sex hormone measures and all-cause mortality in men with esophageal squamous cell carcinoma

Supplementary Figure 1. Heatmap of pairwise Spearman correlation coefficients among preoperative circulating sex hormone measures in men with esophageal squamous cell carcinoma undergoing curative surgery

Supplementary Table 1. Distributions of preoperative circulating levels of sex hormone measures in men with esophageal squamous cell carcinoma

Sex hormones	Number of participants	Mean	Standard deviation	Lower quartile	Median	Upper quartile	Minimum	Maximum
Sex hormone-binding globulin, nmol/L	377	48.96	21.14	35.30	45.70	58.45	9.30	161.20
Testosterone, nmol/L	399	18.58	9.59	11.35	18.68	24.72	1.43	57.44
Free testosterone index	374	3.90	1.69	2.68	3.89	4.85	0.46	9.87
Dehydroepiandrosterone sulfate, μg/dL	398	148.52	76.57	96.30	131.75	188.80	14.80	457.10
Estradiol, nmol/L	352	0.13	0.04	0.11	0.13	0.15	<0.04	0.45
Free estradiol index	330	0.03	0.02	0.02	0.03	0.04	0.01	0.18
Testosterone:estradiol ratio	349	143.67	77.36	86.04	145.28	185.76	6.72	492.51
Prolactin, ng/mL	383	17.60	13.29	10.55	13.74	19.56	2.63	108.24
Follicle-stimulating hormone, mIU/mL	394	10.54	8.10	5.77	8.28	12.23	1.63	59.10
Luteinizing hormone, mIU/mL	393	5.93	3.52	3.55	5.24	7.26	0.89	26.90
Progesterone, ng/mL	377	0.22	0.17	0.11	0.16	0.22	<0.10	2.31

Supplementary Table 2. Paired *t*-tests comparing ln-transformed preoperative and postoperative circulating levels of sex hormone measures in 53 men with esophageal squamous cell carcinoma

Sex hormones	Mean difference[a]	95% CI	*P* value
Sex hormone-binding globulin, nmol/L	-0.219	-0.312, -0.126	<0.001
Testosterone, nmol/L	-0.712	-0.895, -0.528	<0.001
Free testosterone index	-0.492	-0.646, -0.339	<0.001
Dehydroepiandrosterone sulfate, μg/dL	0.027	-0.103, 0.157	0.674
Estradiol, nmol/L	0.189	0.093, 0.286	<0.001
Free estradiol index	0.396	0.244, 0.548	<0.001
Testosterone:estradiol ratio	-0.897	-1.142, -0.652	<0.001
Prolactin, ng/mL	0.151	0.028, 0.274	0.017
Follicle-stimulating hormone, mIU/mL	-0.033	-0.131, 0.066	0.507
Luteinizing hormone, mIU/mL	-0.021	-0.158, 0.115	0.756
Progesterone, ng/mL	0.054	-0.096, 0.204	0.469

[a] Mean difference = mean of [ln(postoperative) − ln(preoperative)]. Positive values indicate higher postoperative levels. CI: confidence interval.

Supplementary Table 3. Sensitivity analyses of associations between preoperative circulating levels of sex hormone measures and all-cause mortality in men with esophageal squamous cell carcinoma

Sex hormone	Number of participants	HR (95% CI)[a]	HR (95% CI)[b]	HR (95% CI)[c]
Sex hormone-binding globulin, nmol/L				
<35.30	94	1.00 (reference)	1.00 (reference)	1.00 (reference)
35.30 to <45.70	93	1.47 (0.96, 2.25)	1.47 (0.96, 2.24)	1.56 (1.06, 2.30)
45.70 to <58.45	96	1.40 (0.91, 2.14)	1.41 (0.92, 2.17)	1.38 (0.93, 2.05)
≥58.45	94	1.29 (0.83, 2.00)	1.18 (0.76, 1.84)	1.49 (1.00, 2.22)
P for trend		0.339	0.523	0.081
Continuous, per 1-SD increase[d]	377	1.14 (0.98, 1.32)	1.12 (0.96, 1.30)	1.16 (1.01, 1.33)
Testosterone, nmol/L				
<11.35	99	1.00 (reference)	1.00 (reference)	1.00 (reference)
11.35 to <18.68	100	0.96 (0.64, 1.45)	0.92 (0.61, 1.39)	0.94 (0.66, 1.35)
18.68 to <24.72	100	0.83 (0.55, 1.26)	0.84 (0.56, 1.27)	0.83 (0.58, 1.20)
≥24.72	100	1.02 (0.67, 1.53)	1.03 (0.68, 1.55)	0.94 (0.65, 1.35)
P for trend		0.873	0.944	0.652
Continuous, per 1-SD increase[d]	399	1.01 (0.87, 1.17)	1.02 (0.88, 1.18)	0.98 (0.85, 1.11)
Free testosterone index				
<2.68	93	1.00 (reference)	1.00 (reference)	1.00 (reference)
2.68 to <3.89	94	0.92 (0.61, 1.39)	0.91 (0.60, 1.38)	0.84 (0.58, 1.21)
3.89 to <4.85	94	0.76 (0.50, 1.15)	0.81 (0.53, 1.23)	0.66 (0.45, 0.97)
≥4.85	93	0.74 (0.47, 1.15)	0.75 (0.48, 1.17)	0.66 (0.45, 0.98)
P for trend		0.116	0.124	0.013
Continuous, per 1-SD increase[d]	374	0.88 (0.76, 1.02)	0.87 (0.75, 1.02)	0.87 (0.76, 0.99)
Dehydroepiandrosterone sulfate, μg/dL				
<96.30	99	1.00 (reference)	1.00 (reference)	1.00 (reference)

96.30 to <131.75	100	0.98 (0.66, 1.46)	1.00 (0.67, 1.48)	0.89 (0.63, 1.26)
131.75 to <188.80	100	1.29 (0.87, 1.91)	1.16 (0.78, 1.72)	1.23 (0.87, 1.75)
≥188.80	99	0.82 (0.53, 1.26)	0.83 (0.53, 1.28)	0.77 (0.52, 1.12)
P for trend		0.702	0.597	0.442
Continuous, per 1-SD increase[d]	398	0.94 (0.81, 1.09)	0.94 (0.81, 1.09)	0.93 (0.82, 1.06)
Estradiol, nmol/L				
<0.106	78	1.00 (reference)	1.00 (reference)	1.00 (reference)
0.106 to <0.132	95	0.82 (0.53, 1.26)	0.89 (0.57, 1.38)	0.74 (0.51, 1.08)
0.132 to <0.154	88	0.81 (0.51, 1.28)	0.85 (0.54, 1.34)	0.75 (0.50, 1.11)
≥0.154	91	0.82 (0.52, 1.28)	0.85 (0.54, 1.33)	0.74 (0.50, 1.09)
P for trend		0.434	0.941	0.585
Continuous, per 1-SD increase[d]	352	0.95 (0.81, 1.12)	0.95 (0.81, 1.12)	0.91 (0.79, 1.05)
Free estradiol index				
<0.021	83	1.00 (reference)	1.00 (reference)	1.00 (reference)
0.021 to <0.028	83	1.02 (0.66, 1.58)	1.11 (0.71, 1.72)	0.77 (0.53, 1.13)
0.028 to <0.038	81	1.05 (0.68, 1.65)	1.22 (0.78, 1.90)	0.76 (0.51, 1.13)
≥0.038	83	0.74 (0.46, 1.20)	0.84 (0.52, 1.36)	0.61 (0.40, 0.92)
P for trend		0.274	0.618	0.025
Continuous, per 1-SD increase[d]	330	0.89 (0.75, 1.05)	0.94 (0.79, 1.10)	0.82 (0.70, 0.95)
Testosterone:estradiol ratio				
<86.04	87	1.00 (reference)	1.00 (reference)	1.00 (reference)
86.04 to <145.28	87	0.98 (0.64, 1.52)	0.99 (0.64, 1.53)	0.96 (0.65, 1.41)
145.28 to <185.76	88	0.76 (0.49, 1.18)	0.81 (0.53, 1.26)	0.76 (0.52, 1.13)
≥185.76	87	0.85 (0.53, 1.35)	0.85 (0.54, 1.34)	0.89 (0.60, 1.33)
P for trend		0.284	0.293	0.303
Continuous, per 1-SD increase[d]	349	1.01 (0.86, 1.19)	1.02 (0.87, 1.19)	1.00 (0.87, 1.15)

Prolactin, ng/mL				
<10.55	95	1.00 (reference)	1.00 (reference)	1.00 (reference)
10.55 to <13.74	96	1.22 (0.80, 1.87)	1.24 (0.81, 1.90)	1.09 (0.75, 1.58)
13.74 to <19.56	96	1.00 (0.65, 1.56)	0.99 (0.64, 1.55)	1.06 (0.73, 1.55)
≥19.56	96	1.50 (0.99, 2.28)	1.49 (0.98, 2.27)	1.34 (0.93, 1.94)
P for trend		0.122	0.130	0.154
Continuous, per 1-SD increase[d]	383	1.14 (0.99, 1.31)	1.14 (0.99, 1.31)	1.12 (0.99, 1.27)
Follicle-stimulating hormone, mIU/mL				
<5.77	98	1.00 (reference)	1.00 (reference)	1.00 (reference)
5.77 to <8.28	99	1.10 (0.73, 1.67)	1.10 (0.72, 1.66)	1.08 (0.75, 1.56)
8.28 to <12.23	99	1.13 (0.75, 1.69)	1.16 (0.77, 1.74)	1.09 (0.76, 1.56)
≥12.23	98	0.83 (0.53, 1.28)	0.83 (0.53, 1.29)	0.82 (0.56, 1.21)
P for trend		0.441	0.426	0.330
Continuous, per 1-SD increase[d]	394	0.96 (0.82, 1.11)	0.97 (0.83, 1.13)	0.94 (0.82, 1.08)
Luteinizing hormone, mIU/mL				
<3.55	98	1.00 (reference)	1.00 (reference)	1.00 (reference)
3.55 to <5.24	97	0.82 (0.54, 1.25)	0.75 (0.49, 1.14)	0.87 (0.60, 1.26)
5.24 to <7.26	100	0.98 (0.66, 1.46)	0.97 (0.65, 1.43)	0.96 (0.68, 1.37)
≥7.26	98	0.82 (0.54, 1.24)	0.79 (0.52, 1.20)	0.80 (0.55, 1.16)
P for trend		0.534	0.584	0.358
Continuous, per 1-SD increase[d]	393	0.90 (0.78, 1.04)	0.90 (0.78, 1.04)	0.90 (0.79, 1.02)
Progesterone, ng/mL				
<0.11	104	1.00 (reference)	1.00 (reference)	1.00 (reference)
0.11 to <0.16	88	0.93 (0.61, 1.41)	1.05 (0.70, 1.59)	0.90 (0.63, 1.29)
0.16 to <0.22	97	1.49 (0.99, 2.25)	1.56 (1.03, 2.35)	1.27 (0.88, 1.84)
≥0.22	88	1.12 (0.73, 1.71)	1.19 (0.78, 1.81)	1.02 (0.70, 1.49)

P for trend		0.244	0.382	0.191
Continuous, per 1-SD increase[d]	377	1.09 (0.93, 1.28)	1.08 (0.92, 1.27)	1.08 (0.93, 1.24)

CI: confidence interval; HR: hazard ratio.

[a] Treat participants lost to follow-up as censored on the date of last contact through telephone follow-up.

[b] Treat all participants lost to follow-up as alive by the end of the study period (May 1, 2023).

[c] Treat all participants lost to follow-up as dead on the date of last contact through telephone follow-up.

[d] Hazard ratio per one standard deviation (SD) increase in ln-transformed hormone levels.

Note: all models adjusted for age and calendar year of surgery, educational level, tobacco smoking, alcohol consumption, pathologic tumor stage, in-hospital postoperative complications, and chemo(radio)therapy.

P for trend was derived by modelling quartile rank (1-4) as a continuous variable.

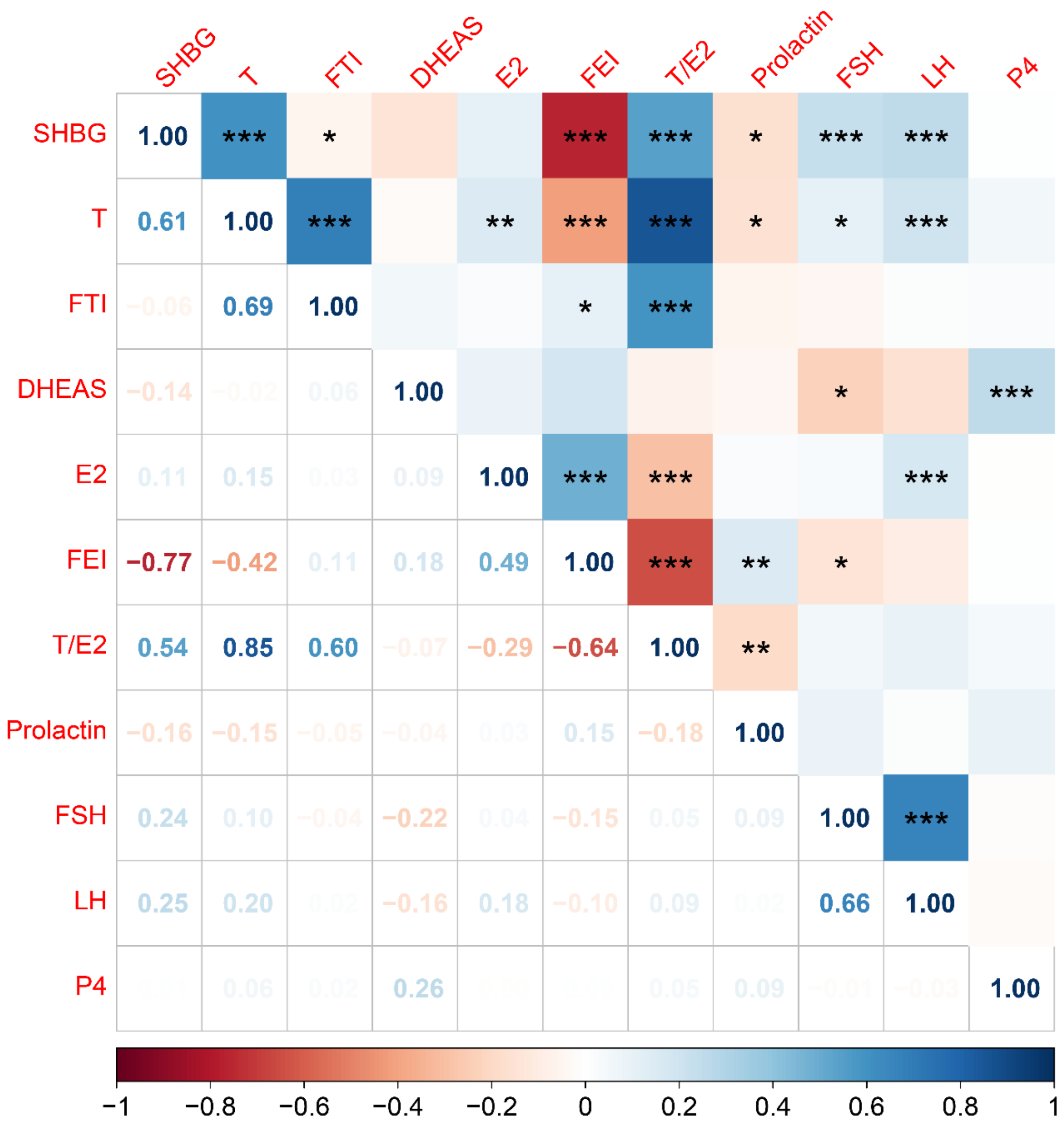

Supplementary Figure 1. Heatmap of pairwise Spearman correlation coefficients among preoperative circulating sex hormone measures in men with esophageal squamous cell carcinoma undergoing curative surgery. SHBG, sex hormone-binding globulin; T, testosterone; FTI, free testosterone index; DHEAS, dehydroepiandrosterone sulfate; E2, estradiol; FEI, free estradiol index; T/E2, testosterone:estradiol ratio; FSH, follicle-stimulating hormone; LH, luteinizing hormone; P4, progesterone. Cells display Spearman's ρ values; asterisks indicate statistical significance (* P<0.05, ** P<0.01, *** P<0.001).
